# From data neglect to data respect: A framework for institutionalising a sustainable research data management culture in Kenyan universities

**DOI:** 10.1371/journal.pone.0359397

**Published:** 2026-11-05

**Authors:** Joel Nakitare, Salome Mathangani, Grace Kamau

**Affiliations:** 1 University Library Services Department, Rongo University, Migori, Kenya; 2 Department of Information Science, Technical University of Kenya, Nairobi, Kenya; University of Namibia, NAMIBIA

## Abstract

Research Data Management (RDM) is increasingly recognised as critical for enhancing research integrity, efficiency, and data reuse globally; however, in Kenya, institutionalised RDM frameworks remain underdeveloped, resulting in fragmented practices, data loss risks, and limited data sharing and reuse. This study, drawn from a PhD investigation, proposes a comprehensive RDM framework informed by empirical evidence on current practices, enablers, and challenges in selected Kenyan universities. The study is anchored on the Australian Research Data Commons (ARDC) Capability Maturity Model for RDM and the UK Data Archive Research Data Lifecycle model. The framework was developed through a multi-stage mixed-methods study comprising a systematic review, a bibliometric analysis, and surveys examining the RDM landscape in Kenyan universities. A multiple-case study design involving six public and private universities, purposively selected based on the June 2023 Webometrics rankings, was adopted. Data was collected through interviews with six head librarians and five heads of research units, as well as questionnaires administered to 306 respondents from a targeted sample of 355. Quantitative data was analysed using IBM SPSS (Version 28), while qualitative data were thematically analysed using NVivo (Version 14). Triangulation and pretesting were used to enhance validity and reliability. All necessary ethical considerations were addressed before data collection. Findings indicate a growing awareness of RDM among researchers; however, practices remain largely ad hoc, poorly coordinated, and weakly supported institutionally. Notably, none of the universities studied had dedicated RDM policies, structured RDM support services, or dedicated RDM Information and Communication Technology (ICT) infrastructure, revealing gaps across personal, institutional, and national levels. This study proposes a multi-level, stakeholder-driven RDM framework that integrates researchers, librarians, ICT personnel, research offices, and key national agencies such as the National Commission for Science, Technology and Innovation (NACOSTI), the Commission for University Education (CUE), and the Office of the Data Protection Commissioner (ODPC) to support coordinated, policy-driven data stewardship aligned with international best practices for sustainable RDM in Kenyan universities.

## Introduction

In the contemporary knowledge economy, research data has emerged as one of the most strategically valuable assets in science, innovation, and societal development. Beyond being a mere by-product of research activities, data now constitutes a critical intellectual resource that embodies substantial investments in expertise, time, infrastructure, and funding [1]. Its value extends beyond the lifespan of the original study by enabling verification, reproducibility, secondary analysis, interdisciplinary collaboration, and new scientific discovery [2]. Increasingly, research data is being likened to “*the new oil*” because of its transformative potential when systematically curated, shared, and analyzed to generate knowledge and inform policy decisions [3]. Moreover, in the era of Artificial Intelligence (AI), high-quality and trustworthy research data has become the foundational currency powering machine learning systems, digital innovation, and evidence-based decision-making [4]. As [5] observe, even the most sophisticated AI technologies remain unreliable and unsustainable without clean, credible, and well-governed data. Consequently, the effective management, stewardship, accessibility, and preservation of research data are no longer optional institutional practices but essential components of responsible and impactful scholarship. Without robust Research Data Management (RDM) frameworks, scientific transparency is weakened, reproducibility becomes difficult, and the integrity of the broader knowledge production ecosystem is fundamentally compromised [6].

Globally, the evolution of RDM practices reflects a transition from historically fragmented and poorly coordinated data practices towards increasingly structured and policy-driven approaches to research data stewardship. Before the 2000s, limited attention was given to the long-term management, preservation, and reuse of research data, resulting in substantial volumes of valuable data being distorted, underutilised, or permanently lost before their full scientific potential could be realized [7,8]. However, the emergence of the “data deluge” in the early 21st century, driven by increased stakeholder demand, fundamentally transformed the RDM landscape [9–11]. To date, many developed countries have increasingly institutionalized RDM through formal frameworks, policies, and infrastructures, including mandatory Data Management Plans (DMPs), institutional repositories, open data mandates, and specialized roles such as data librarians and data stewards [7,12]. The growing attention to RDM by governments, institutions, and academic disciplines has led to the emergence of diverse RDM frameworks, including institutional frameworks focused on governance and compliance [13], disciplinary frameworks designed to address domain-specific data needs, and international initiatives such as the FAIR Principles and the European Open Science Cloud (EOSC), which aim to promote harmonized and interoperable global data practices [14,15]. Countries such as the United Kingdom, the United States, Canada, and Australia have consequently established mature national RDM strategies to support responsible data governance across research institutions [7,16,17].

Despite significant global advancements in RDM, particularly in developed countries, substantial disparities persist across regions, institutions, and disciplines due to differences in policy environments, institutional capacities, disciplinary cultures, and researcher competencies [16,18]. In Africa, RDM development remains relatively underdeveloped, with limited evidence of coordinated RDM practices across most countries [19,20]. South Africa remains a notable exception, having made considerable progress in establishing national policies, institutional repositories, and structured RDM initiatives. In contrast, many African countries still lack formal national RDM strategies and institutional mechanisms to support effective research data governance. Consequently, RDM practices in many African countries remain fragmented, inconsistent, and largely institution-specific, characterised by disjointed institutional efforts, unclear legal and ethical frameworks, inadequate investment in research data infrastructure, and weak coordination of data stewardship practices [21–23]. These persistent gaps highlight the need for comprehensive national RDM frameworks that align with global best practices while strengthening the culture of RDM and institutional competitiveness across the African research ecosystem.

### State of RDM practices in universities

According to [31,32], RDM practices in universities have become increasingly institutionalised in developed countries, where they are supported by comprehensive policy frameworks, dedicated technological infrastructure, specialised support services, and strong national mandates. As such, universities in countries such as the United Kingdom, Australia, the Netherlands, and the United States have embedded RDM into institutional research governance through mandatory data management plans, institutional repositories, data stewardship programmes, dedicated research data services within libraries, and alignment with the FAIR data principles [24,25]. These mature RDM ecosystems are further reinforced by national funding agencies and research policies that require responsible data management and promote open science [26].

In contrast, RDM implementation in many developing countries remains in its infancy, characterised by the absence of institutional policies, limited technological infrastructure, inadequate funding, and insufficient human capacity [21,27,28]. Although pockets of good practice exist at the personal level, these efforts remain isolated [29–31]. Consequently, RDM practices in these countries are largely individualised, with researchers relying on personal storage devices, self- developed data management approaches, and informal data sharing practices rather than institutionally coordinated systems [20,28,32]. This contrast suggests that while developed countries have largely transitioned from researcher- centred to institutionally embedded RDM systems, universities in developing countries are still in the early stages of building the policies, infrastructure, governance structures, and human capacity necessary to establish sustainable and coordinated RDM ecosystems.

Evidence from the literature shows that barriers to effective RDM in universities are multidimensional and operate at the individual, institutional, and national levels, with these factors often reinforcing one another. At the individual level, the stakeholders frequently lack the knowledge, technical competencies, and data literacy required to manage research data throughout its lifecycle, resulting in poor documentation, inadequate metadata, weak data preservation, and limited data sharing and reuse [33]. At the institutional level, universities often face the absence of RDM policies, inadequate ICT infrastructure, a lack of dedicated RDM support services, and insufficient funding, among other issues, leading to fragmented and inconsistent RDM implementation [25,27,28]. At the national level, the absence of comprehensive RDM policies, weak coordination among research regulatory agencies, limited funding mechanisms, and the lack of national standards for research data governance further constrain institutional implementation [10,21,26]. As such, the literature demonstrates that effective RDM implementation requires coordinated interventions across all three levels.

### Context of the study

Kenya’s research ecosystem is characterized by growing institutional capacity, increasing research productivity, expanding postgraduate enrolment, and a steadily growing open science movement supported by open-access repositories [34]. According to [35], Kenya's higher education sector has expanded significantly over the past two decades. However, disparities in funding, research output, student enrolment, and academic staffing persist, with men continuing to constitute the majority of students and academic staff. Research is a core mandate of Kenyan universities, most of which have established Research Divisions or Directorates, supported by research administrators, librarians, ICT personnel, grant officers, and ethics review committees to coordinate and oversee research activities. As such, Universities continuously generate substantial volumes of research data across diverse disciplines, with more than 5,843 research projects registered annually during the 2020/2021 financial year [36]. Research activities in universities are regulated through the CUE, which requires universities to establish mechanisms for managing and disseminating research outputs as part of quality assurance and institutional accreditation processes, and the NACOSTI, whose ethical review and research authorization frameworks emphasize research compliance, accountability, and ethical conduct [29,37]. Despite this elaborate and highly regulated research environment, limited attention has been paid to structured RDM. Existing regulatory and institutional frameworks predominantly focus on research ethics, publications, and dissemination of outputs, with minimal emphasis on policies and systems for the stewardship, curation, preservation, sharing, and long-term management of research data, thereby revealing a significant policy and practice gap within Kenya’s research ecosystem.

### Theoretical frameworks

The study was guided by two complementary frameworks: the UK Data Archive Research Data Lifecycle Model and the Australian Research Data Commons (ARDC) Capability Maturity Model for Research Data Management (RDM) [38–40]. The integration of these models provided both a process-oriented and an evaluative perspective for examining RDM practices within the research environment. The ARDC Capability Maturity Model was used as the primary framework for assessing the maturity of RDM practices and institutional readiness because it provides a structured, staged approach to evaluating the extent to which RDM capabilities are developed across an institution [38,39]. The maturity framework further informed the development of the study variables and indicators by providing measurable criteria for evaluating RDM capacity and the adoption of practices [38,41]. The UK Data Archive Research Data Lifecycle Model was used to complement the ARDC, as it conceptualises RDM as a continuous process spanning data creation, processing, documentation, storage, preservation, sharing, reuse, and disposal [32,42]. As such, it enabled the study to identify data-intensive stages in which researchers face the greatest challenges and institutional support mechanisms are most critical. The combined application of the two frameworks strengthened the study's theoretical foundation by linking institutional RDM capability to practical data management activities throughout the research lifecycle.

### Rationale of the study

Kenya’s research landscape is evolving, with growing recognition of the importance of RDM. Despite this progress, effective RDM practices remain limited within Kenyan universities. Notably, the Directory of Data Repositories (Re3data.org, 2024) lists only five repositories in Kenya, none of which are affiliated with a university. Additionally, national funding bodies, such as the National Research Fund (NRF), do not require data management plans, unlike many international funders. Several factors contribute to this gap, including low data literacy among researchers, inadequate infrastructure, and a lack of integrated policies and support services [29,30,37,43,44]. As a result, research data is often stored on personal devices or external platforms, exposing it to loss, ethical risks, and reduced credibility. This fragmented approach undermines the quality, sustainability, and global competitiveness of research outcomes. To address these issues, a structured RDM framework needs to be tailored to the context of Kenyan higher education. This study proposes a multi-level framework that, if adopted, can support the institutionalization of a sustainable RDM culture in Kenyan universities.

### Objectives of the study

The primary objective of the study was to examine current research data management practices at selected universities in Kenya and to propose a framework to enhance research outcomes at these universities. The following specific objectives guided the study:

1. To determine the current research data management practices in universities in Kenya.
2. To analyse the state of the research data management enablers in universities in Kenya.
3. To examine the challenges affecting effective research data management practices in Kenyan universities.
4. To propose a framework that can enhance research outcomes in these universities.

## Methodology

The framework was based on a multi-stage mixed-methods approach that enabled the collection and integration of quantitative and qualitative data. It combined a systematic review of early indicators, bibliometric analysis, and a survey to provide a comprehensive understanding of the RDM landscape in Kenyan universities. This study was grounded in a pragmatist philosophical worldview, which facilitates the integration of quantitative and qualitative paradigms [45]. Given that RDM is an emerging, multidisciplinary field that involves multiple stakeholders, a mixed-methods design was employed to leverage methodological pluralism and achieve triangulation of findings [46].

The study used a multi-stage, mixed-methods research design comprising a web content analysis of early indicators of RDM in the country, an informetric analysis of Kenyan datasets archived in the Dimensions database, and empirical surveys that analysed primary data collected from six case studies. For the scoping review, the researcher defined inclusion and exclusion criteria to retrieve relevant literature such as the 2010 Constitution of Kenya, Acts of Parliament, reports, and policies from databases, institutional repositories, official websites, and grey literature sources, while for the survey, primary data was collected from the selected respondents.

The study population comprised all universities in Kenya, while the target population consisted of all accredited public and private universities engaged in postgraduate teaching and research. From the target population, six universities (three public and three private) were purposively selected for multiple studies based on the June 2023 Webometrics ranking, institutional age, size, and disciplinary diversity. To ensure a comprehensive understanding of RDM within the selected cases, multiple sampling techniques were employed. Respondents were categorized by their roles to capture a holistic view of the research ecosystem. Academic staff and postgraduate students formed the population from which respondents were drawn to assess RDM practices. Purposive sampling was used to select the heads of libraries and research units in the selected universities. These served as key informants for the study due to their direct involvement in RDM processes. Given their small number, census sampling was applied to all six Heads of research and library units. For academic staff and postgraduate students, probability sampling was applied. Krejcie and Morgan’s (1970) formula was used to determine a sample size of 378 respondents, which was proportionally allocated into 53 academic staff and 325 postgraduate students across the selected universities. Within each stratum, simple random sampling was used to select individual participants, ensuring equal probability of selection and minimizing sampling bias.

Data were collected using questionnaires for academic staff and postgraduate students, while qualitative data were obtained through interviews with Heads of Libraries and Heads of Research Units. In addition, a content analysis of selected national legal instruments and institutional research-related policies was conducted to enable comparison, contextual interpretation, and triangulation of findings (Yin, 2014). Quantitative data were analysed using IBM SPSS (Version 28), while qualitative data were thematically analysed using NVivo (Version 14). Before data collection, all necessary ethical approvals, permissions, and informed consent were obtained. Interview participants provided written informed consent, while questionnaire respondents signed and dated consent forms after reviewing the study information sheet. Data were collected between 14 June 2023 and 13 June 2024. Ethical clearance was obtained from NACOSTI (Ref.). No. 261358, and approval was granted by the Daystar University Institutional Scientific and Ethics Review Committee (DU-ISERC/14/June/2023/0088).

## Findings and discussions

This section presents the findings on the RDM landscape in Kenyan universities, drawing on survey, interview, content, and informetric data. The findings triangulate evidence on individual and institutional RDM practices, perceived barriers, and national research data trends. The study achieved high response rates, with 306 of 355 researchers (86.2%), all six head librarians (100%), and five of six heads of research units (83.3%) participating, thereby enhancing the reliability and credibility of the findings [45]. In addition, content analysis of publications, reports, guidelines, and web-based resources on RDM in Kenya, together with an informetric analysis of 45,737 Kenyan-associated datasets in the *Dimensions* database (1952–2023), provided complementary evidence on research data production and archiving practices.

### Demographic characteristics of the respondents

Based on Table 1, the study revealed a gender distribution of 60.5% male and 39.5% female participants. In terms of institutional affiliation, 58.2% were from public universities and 41.8% from private universities. The frequency of research engagement varied: 37.3% of respondents engaged annually, 29.1% monthly, and 14.7% daily. Participants held a range of academic and research titles, with lecturers (22.2%), senior lecturers (21.9%), and master’s candidates (20.3%) comprising the majority, followed by assistant lecturers (18.6%), PhD candidates (11.1%), and professors (5.9%). This distribution indicates that most respondents were actively engaged in research. Regarding experience, 33% of respondents reported 6–10 years of research involvement, 25.5% had 11–15 years, and 24.8% had less than 5 years. Smaller proportions had 16–20 years (9.5%), 21–25 years (2.6%), and more than 26 years (4.6%) of research experience. Disciplinary representation was also diverse, with the largest groups in education, counselling, and psychology (17.3%), business and economics (15.7%), and health sciences (12.7%). These findings highlight broad disciplinary participation and emphasise active engagement with RDM across Kenyan universities.

**Table 1:** Demographic characteristics of the respondents.

Socio-demographic Features		Frequency	Per cent
Category of the University	Public	178	58.2
	Private	128	41.8
	**Total**	**306**	**100**
Gender	Male	185	60.5
	Female	121	39.5
	**Total**	**306**	**100**
Title of the Respondents	Professor	18	5.9
	Senior Lecturer	67	21.9
	Lecturer	68	22.2
	Assistant Lecturer	57	18.6
	PhD candidate	34	11.1
	Masters candidate	62	20.3
	**Total**	**306**	**100**
Discipline	Biological and physical sciences	28	9.2
	Education, Counselling & Psychology	53	17.3
	Information, Computing & IT	63	20.6
	Health Sciences	39	12.7
	Business, Hospitality & Economics	48	15.7
	Engineering & Technology	9	2.9
	Agriculture & Environmental Science	12	3.9
	Arts, Humanities & Social Sciences	54	17.6
	**Total**	**306**	**100**
Experience in Research	0-5	76	24.8
	6-10	101	33.0
	11-15	78	25.5
	16-20	29	9.5
	21-25	8	2.6
	over26	14	4.6
	**Total**	**306**	**100**
Frequency of involvement in research	Daily	45	14.7
	Weekly	45	14.7
	Monthly	89	29.1
	Yearly	114	37.3
	Rarely	13	4.2
	**Total**	**306**	**100**

### The state of RDM practices at the individual level

The Survey and interview data revealed that RDM practices in Kenyan universities are fragmented, largely informal, and driven primarily by individual initiative rather than institutional frameworks. From the data, although the majority of researchers, 207 (67.6%), indicated that they utilize IT tools in collection and documentation, formalized data planning remains limited. Over half of the respondents, 155 (50.6%), do not employ formal strategies to ensure data quality, and 161 (62.4%) do not maintain data diaries or Data Management Plans (DMPs).

Furthermore, manual documentation practices remain unstandardized, with researchers almost evenly split, 161 (52.6%), on the maintenance of research diaries. At the institutional level, structural support for comprehensive RDM is minimal. Responsibilities are largely relegated to individual researchers or student-supervisor dyads. Although universities enforce basic quality controls through supervisory assignments, proposal guidelines, and thesis examinations, none mandate the submission of a DMP, underscoring a significant institutional and national gap in standardizing verifiable research data workflows.

Regarding data organization and storage, Table 2 shows that the majority, 260 (85.0%), enforced a defined file-naming and organization system, while 290 (94.8%) indicated they store data in multiple locations. Data from interviews corroborated the widespread use of multiple storage locations as a proactive, ad hoc safeguarding strategy due to a lack of university repositories for archiving raw research data. Despite this high awareness of file organization, only a slight majority of respondents, 166 (54.2%), maintained regular data backup schedules, leaving a substantial 140 (45.7%) vulnerable to data loss due to a lack of routine backup protocols. A similar pattern was observed in data preservation and archiving practices, as the vast majority of researchers, 276 (90.2%), said they did not archive their data on university servers or repositories; instead, 246 (80.4%) of researchers relied on personal emails, USB drives, external hard drives, and personal computers to preserve their datasets. Interviews with institutional librarians and heads of research units revealed consensus: none of the sampled universities had a dedicated research data repository. Regarding data sharing and accessibility practices, a majority of 236 (77.1%) respondents indicated that they don’t mind sharing their data, while 197 (64.4%) indicated that they actively reuse existing secondary datasets. However, this willingness did not translate into data sharing, as 234 (76.4%) respondents reported not actively sharing their data. Similarly, 242 (79.1%) have not signed up for any data-sharing platforms such as Figshare, GBIF, or Zenodo.

**Table 2:** Research data management practices among researchers.

Data planning practices				
	Strongly Disagree	Disagree	Agree	Strongly Agree
I put in place formal strategies to get quality and verifiable data	42 (13.7%)	113 (36.9%)	107 (35.0%)	44 (14.4%)
I prepare data management plans, datasets, and their associated metadata.	50 (16.3%)	141 (46.1%)	88 (28.8%)	27(8.8%)
**Data collection & documentation practices**				
I keep a diary of my data project	42 (13.7%)	103 (33.7%)	108 (35.3%)	53 (17.3%)
I use IT tools to collect data	39 (12.7%)	60 (19.6%)	136 (44.4%)	71 (23.2%)
**Data organisation and Storage practices**				
I have a defined way of organising and naming data files	11(3.6%)	35 (11.4%)	168 (54.9%)	92(30.1%)
I keep my data in multiple places, like email, USB, and pc	6(2.0%)	10(3.3%)	84(27.5%)	206(67.3%)
I regularly back up my research data and backup schedules	68(22.2%)	72(23.5%)	98(32.0%)	68(22.2%)
**Data preservation and archiving practices**				
I preserve/ archive my data in the university server/repository	144(47.1%)	132(43.1%)	21(6.9%)	9(2.9%)
I preserve/ archive on email or secondary devices	25(8.2%)	35(11.4%)	100(32.7%)	146(47.7%)
I preserve/ archive my unused data for the future	32(10.5%)	36(11.8%)	118(38.6%)	120(39.2%)
I preserve/ archive my data in online data repositories	141(46.1%)	120(39.2%)	29(9.5%)	16(5.2%)
**Data sharing and accessibility practices**				
I do not mind sharing my data with other stakeholders	23(7.5%)	47(15.4%)	131(42.8%)	105(34.3%)
I use other people's data	39(12.7%)	70(22.9%)	111(36.3)	86(28.1%)
I frequently share my data by linking to the publication	68(22.2%)	166(54.2%)	58(19.0%)	14(4.6%)
I am a member of data-sharing platforms like the Global Biodiversity Information Facility (GBIF)	122(39.9%)	120(39.2%)	38(12.4)	26(8.5%)

The scoping review of literature established that RDM practices in Kenya occur only minimally at the individual researcher's level, creating a fragmented national landscape. This lack of cohesion is further compounded by regulatory omissions; for instance, the scoping review revealed that all the funding calls of the National Research Fund, Kenya, do not explicitly require grant applicants to submit Data Management Plans (DMPs), as is the practice of other funders. The informetric analysis similarly revealed a disjointed pattern in which most university-based researchers rarely use their own institutional repositories to preserve data, despite demonstrating a high level of awareness of the overall importance of preserving scholarly content. The analysis found that among the top 100 individual researchers contributing most to the Kenya-related datasets pool, there is a perfectly balanced split: 50% (50 researchers) are affiliated with Kenyan institutions and the other 50% with organizations outside the country. However, when evaluating the total volume of archived data, the informetric analysis found that non-Kenyan researchers and foreign institutions manage slightly more than their local counterparts, specifically archiving 53% of the datasets, compared to 47% archived by local Kenyan researchers. Among these international contributors, the informetric analysis determined that the absolute majority are based in the United States, accounting for 30% of the datasets generated by 28 researchers, followed by the United Kingdom, which accounts for 11% of the datasets generated by 12 researchers.

### The state of research data management at the university level

The findings are presented according to four key indicators of the Capability Maturity Model: RDM legal and policy frameworks, RDM ICT infrastructure, and RDM support services. Quantitative survey data from researchers (Table 3) are integrated with qualitative insights from the reviewed literature.

**Table 3:** State of research data management enablers in Kenyan universities.

RDM Policies and Guidelines in the University				
	Strongly Disagree	Disagree	Agree	Strongly Agree
Adequate/suitable RDM policies and guidelines in the University	62(20.3%)	128(41.8%)	83(27.1%)	33(10.8%)
Well-communicated RDM policies and guidelines	54(17.6%)	155(50.7%)	68(22.2%)	29(9.5%)
Established procedures for data storage	87(28.4%)	148(48.4%)	44(14.4%)	27(8.8%)
**RDM IT Infrastructure in Universities**				
Robust I.T. Infrastructure	63(20.6%)	108(35.3%)	74(24.2%)	61(19.9%)
Adequate Data Software	55(18.0%)	114(37.3%)	84(27.5%)	53(17.3%)
The university progressively improves ICT facilities	45(14.7%)	106(34.6%)	92(30.1%)	63(20.6%)
**RDM Support Services in Universities**				
The University has adequate support services for researchers	57(18.6%)	142(46.4%)	74(24.2%)	33(10.8%)
Frequent training and guidance	71(23.2%)	99(32.4%)	82(26.8%)	54(17.6%)
Dedicated RDM personnel/office/and data librarian	61(19.9%)	210(68.6%)	24(8.3%)	11(3.1%)

### State of legal frameworks and policies for RDM

The content analysis of the legal instruments shows that, while foundational legislation exists in the Constitution of Kenya (2010) and in a few Acts such as the Kenya Data Protection Act (2019), the Science, Technology, and Innovation Act (2013), and the Universities Act (2012), explicit and comprehensive guidelines dedicated to RDM remain absent at the national level. As a result, the national legal and policy framework for RDM in Kenya is inadequate and fragmented. This statutory deficit has affected institutional compliance; none of the six universities evaluated in the study had successfully implemented a university-level RDM policy. Although five of these universities maintained operational institutional repositories, none were actively archiving raw research data. Furthermore, oversight bodies such as NACOSTI and the Commission for University Education (CUE) do not currently enforce explicit RDM guidelines or mandate Data Management Plans (DMPs) for researchers. Consistent with this gap, the data in Table 3 show that 190 (62.1%) of respondents rated their institutional policies as inadequate, 209 (68.3%) reported poor communication or the absence of policy, and 235 (76.8%) cited unclear data procedures. Interviews further confirmed that only one university had incorporated RDM elements into a revised research policy, but implementation was limited by weak IT infrastructure, limited staff capacity, and high workloads.

### State of IT infrastructure for RDM

The scoping review revealed that most Kenyan research institutions have adequate general ICT infrastructure, such as Hardware, high-speed fibre internet and Institutional repositories; however, the majority don’t have dedicated data archiving platforms. According to the Registry of Research Data Repositories, no Kenyan university has a dedicated data repository. This finding was corroborated by the survey data, which showed that while five of the six studied universities have functioning institutional repositories (IRs) and all six have institutional repository policies, none of these repositories are currently archiving research data. The review also revealed that despite these infrastructural constraints, efforts to preserve research data are gaining momentum, as evidenced by more than 45,000 datasets associated with Kenya deposited in external global databases such as Dimensions, Zenodo, and Figshare.

According to Table 3, the findings on IT infrastructure for RDM highlighted mixed perceptions among stakeholders, with a general trend toward dissatisfaction. Over half of the respondents, 171 (55.9%), considered their institutions’ IT infrastructure inadequate for RDM. A similar divide was observed regarding the adequacy of software tools, as 169 (55.3%) disagreed that their universities provided adequate data software. Despite these gaps, perceptions were slightly more optimistic about the institutional trajectory, with 155 (50.7%) respondents reporting that their university is progressively improving its ICT facilities. However, a substantial 151 (49.3%) slice of the sample felt that facility upgrades remained either stagnant or insufficient, underscoring the urgent need for targeted infrastructural expansion to support complex data lifecycles. As such, this statistical divide aligns with findings that although most universities possessed IT infrastructure such as servers, Wi-Fi networks, institutional repositories, and computer labs, none had dedicated RDM infrastructure such as institutional data repositories or specialized data management software

### State of RDM support services

Based on the reviewed literature, the landscape of RDM support services in Kenya is currently in its infancy and faces significant structural gaps. While general research support services, such as training and tools like a similarity checker and citation software, exist, specialized RDM support services remain severely limited. There is no evidence of dedicated RDM support desks or specialized roles, such as data librarians, within Kenyan academic libraries. This lack of specialized personnel leaves researchers largely on their own. These findings corroborate the survey data, as 65% disagreed with the assertion that universities had adequate support services for researchers. This trend of dissatisfaction extended to the provision of training, where 55.6% disagreed that regular training and guidance on RDM practices are available. When queried about the presence of dedicated RDM personnel, a specialized office, or a data librarian, an overwhelming majority, 88.5% of the stakeholders, reported an absolute absence of these resources. Data from the interviews indicated that libraries provided general research support, including access to databases and repositories, as well as information literacy training, but none offered specialised RDM services.

### Challenges for effective research data management

The study identified challenges to effective RDM at three levels: personal, institutional, and national. The findings indicated that effective RDM in Kenyan universities is severely constrained by low data literacy, procedural gaps, and reliance on fragmented, ad hoc practices at the individual level. A lack of standardized guidelines and training leaves 191 (62.4%) of researchers unable to draft Data Management Plans (DMPs), creating immediate barriers to international funding. This deficiency extends to data creation and security: approximately half of the researchers fail to implement formal quality control strategies 155 (50.6%) or maintain research diaries 145 (47.4%), while an overwhelming 290 (94.8%) store active data across vulnerable, decentralized personal devices without systematic backup routines 140 (45.7%).

Furthermore, a profound disconnect exists between preservation and archiving intent due to insufficient IT infrastructure and capacity. Despite widespread conceptual support for data reuse, 276 (90.2%) of researchers don’t use institutional servers, while 261 (85.3%) don’t use available data repositories such as Zenodo, even though they are freely accessible online. This creates a “silo effect” that fosters a reciprocity paradox, while 197 (64.4%) of researchers consume external data, 234 (76.4%) fail to link their own datasets to scholarly publications due to apprehensions surrounding copyright, ethics, and a lack of localized expert guidance from data librarians.

These institutional challenges are critically compounded by severe infrastructural, technical, and administrative deficits, notably the complete absence of dedicated institutional data repositories across all studied cases. Universities are largely unable to provide adequate storage solutions, advanced processing tools, secure backup facilities, or robust policy and support frameworks. Consequently, research data is managed purely at a personal level, leaving it highly vulnerable to premature loss, distortion, or destruction. This infrastructure and capacity gap ultimately causes local university researchers to lag behind peers in NGOs and dedicated research institutes, increasing their exposure to data loss, minimizing their integration into global open science networks, and creating a heavy reliance on foreign partners that undermines local data ownership and intellectual sovereignty.

These institutional deficiencies are further compounded by structural challenges at the national level, most notably the total absence of a comprehensive national legal and policy framework for research data management. Without a centralised, formalised approach from national regulatory and funding bodies, universities and researchers are forced to operate in isolation. This vacuum results in a severe lack of standardized protocols required to ensure consistency, security, and long-term sustainability in data practices across the higher education sector. Furthermore, this policy omission is mirrored by deep-seated gaps in the national academic curricula, which fail to embed foundational data literacy and modern RDM methodologies into undergraduate and postgraduate training. Consequently, the lack of both top-down national governance and bottom-up educational integration leaves the academic ecosystem poorly equipped to transition toward contemporary open science frameworks.

## Discussion of the findings

The demographic findings show that university education and research in Kenya are predominantly male-dominated and concentrated in public universities. This situation mainly results from higher enrolment rates in public universities, driven by factors such as government funding, lower tuition fees, larger infrastructure, and favourable student placement policies administered by the Kenya Universities and Colleges Central Placement Service (KUCCPS). The gender gap in staffing also reflects wider trends in enrolment and graduation rates, often influenced by cultural factors that restrict women's access to higher education. These findings align with national data from the Commission for University Education, which show a higher proportion of male staff and public university affiliations among academics and researchers [35]. Additionally, RDM practices differ across disciplines, levels of experience, job tiers, and research participation, as noted by [32], who identified discipline-specific RDM groups. Researchers in the health sciences tend to adhere to better practices, likely due to the strict ethical and privacy standards required when handling sensitive data [5]. Despite demographic variations, this study provides a snapshot of RDM practices in Kenyan universities, with wider implications for similar institutions in developing countries.

### The state of RDM practices at the individual level

The findings demonstrate that RDM has yet to become an institutionalised function within the Kenyan universities studied. The absence of coordinated governance structures, formal policies, dedicated infrastructure, and specialised support services limits institutions’ ability to implement consistent and sustainable RDM practices across the research lifecycle. As a result, responsibility for managing research data continues to rest primarily with individual researchers, creating variability in practice and increasing the risk of inconsistent data stewardship. This situation reflects broader challenges reported across many developing countries, where weak institutional governance, inadequate digital infrastructure, limited investment in RDM services, and insufficient organisational capacity continue to impede the transition from researcher-driven data management to institution-wide RDM ecosystems.

Although researcher-led RDM practices recognise the value of research data, their sustainability and effectiveness remain constrained by a lack of institutional coordination, resulting in inconsistent data stewardship and limited opportunities for data sharing, reuse, and compliance with emerging research governance requirements.

### The state of research data management enablers at the university level

On the state of RDM enablers, the findings suggest that the institutional capacity required to support effective RDM remains underdeveloped in the surveyed universities, reflecting low levels of preparedness for institution-wide research data stewardship. The absence of RDM policies, dedicated IT infrastructure and support services, and adequate competencies among researchers limits institutions’ ability to implement systematic RDM across the research lifecycle. This pattern is consistent with evidence from developing countries, where investments have largely focused on general ICT infrastructure rather than purpose-built RDM systems and services [10,33]. Collectively, these findings underscore that sustainable RDM requires a comprehensive framework that coordinates an institutional approach that integrates policy, infrastructure, support services, and capacity development, rather than isolated investments in individual components. This finding aligns with evidence from sub-Saharan Africa, where many universities have yet to institutionalise RDM through formal policies despite growing international expectations for responsible data stewardship and adherence to the FAIR Guiding Principles [21,24].

Viewed through the Capability Maturity Model, these findings indicate that the surveyed universities remain at the lower end of RDM maturity, operating predominantly at Level 1 (Initial) with limited evidence of progression towards Level 2 (Managed). The absence of institutional governance mechanisms, specialised infrastructure, coordinated support services, and adequate human capacity suggests that RDM is neither standardised nor embedded as an organisational capability. Although some foundational ICT infrastructure exists, it is not purpose-built to support the research data lifecycle and therefore contributes only marginally to institutional RDM maturity. Consequently, RDM practices remain largely ad hoc, researcher-dependent, and inconsistently implemented across the institutions. Advancing to higher maturity levels will require establishing an integrated institutional RDM framework that aligns governance, infrastructure, services, and capacity development, transforming isolated practices into standardised, institution-wide processes consistent with international best practices and the FAIR Guiding Principles.

### Challenges for effective research data management

Collectively, the findings indicate that effective RDM in the studied Kenyan universities is constrained by interrelated challenges at the individual, institutional, and national levels. Limited researcher competencies, weak institutional governance, inadequate technological and service infrastructure, and the absence of a coordinated national policy framework have impeded the institutionalisation of RDM as a strategic organisational capability. These interconnected barriers reinforce fragmented and inconsistent data management practices while limiting compliance with open science and FAIR data principles. This pattern is consistent with evidence from developing countries, where insufficient data literacy continues to impede effective RDM despite growing international expectations for responsible stewardship of research data [30,34,47]. These findings highlight that sustainable RDM requires not only technological and policy investments but also systematic capacity-building to develop researchers’ RDM competencies.

These findings highlight the need for stronger national leadership and coordinated RDM governance to promote consistent institutional implementation, strengthen investment in RDM capacity and infrastructure, and align Kenya's research ecosystem with international data stewardship and open science standards. Further, the findings suggest that strengthening RDM in Kenya will require a coordinated, multi-level approach that simultaneously addresses researcher capacity, institutional capability, and national policy and governance, consistent with international evidence that sustainable RDM depends on integrated governance, infrastructure, support services, and skilled human capital [5,12,34,44].

### Recommendations

Based on the findings, the study recommends a comprehensive framework to guide RDM practices in Kenyan universities. Although the framework developed in this study is based on analyzed literature and six case-studied universities, it proposes a strategic pathway for Kenyan universities to adopt robust, scalable, and collaborative RDM practices. By clarifying stakeholder roles and outlining structural interdependencies, the framework provides a practical, actionable model for improving data stewardship and overall research quality. Adopting this framework will enable universities in Kenya to establish a strategic infrastructure for managing research data effectively by identifying key players, assigning clear responsibilities, and aligning them with international best practices. Based on the findings, the study recommends that the universities in Kenya adopt and implement a framework for sustainable RDM culture to elevate their research outcomes and institutional impact. As shown in Fig 1, the framework offers a structured, practical, and sustainable roadmap for advancing effective RDM practices within Kenya’s higher education sector.

**Fig 1:**
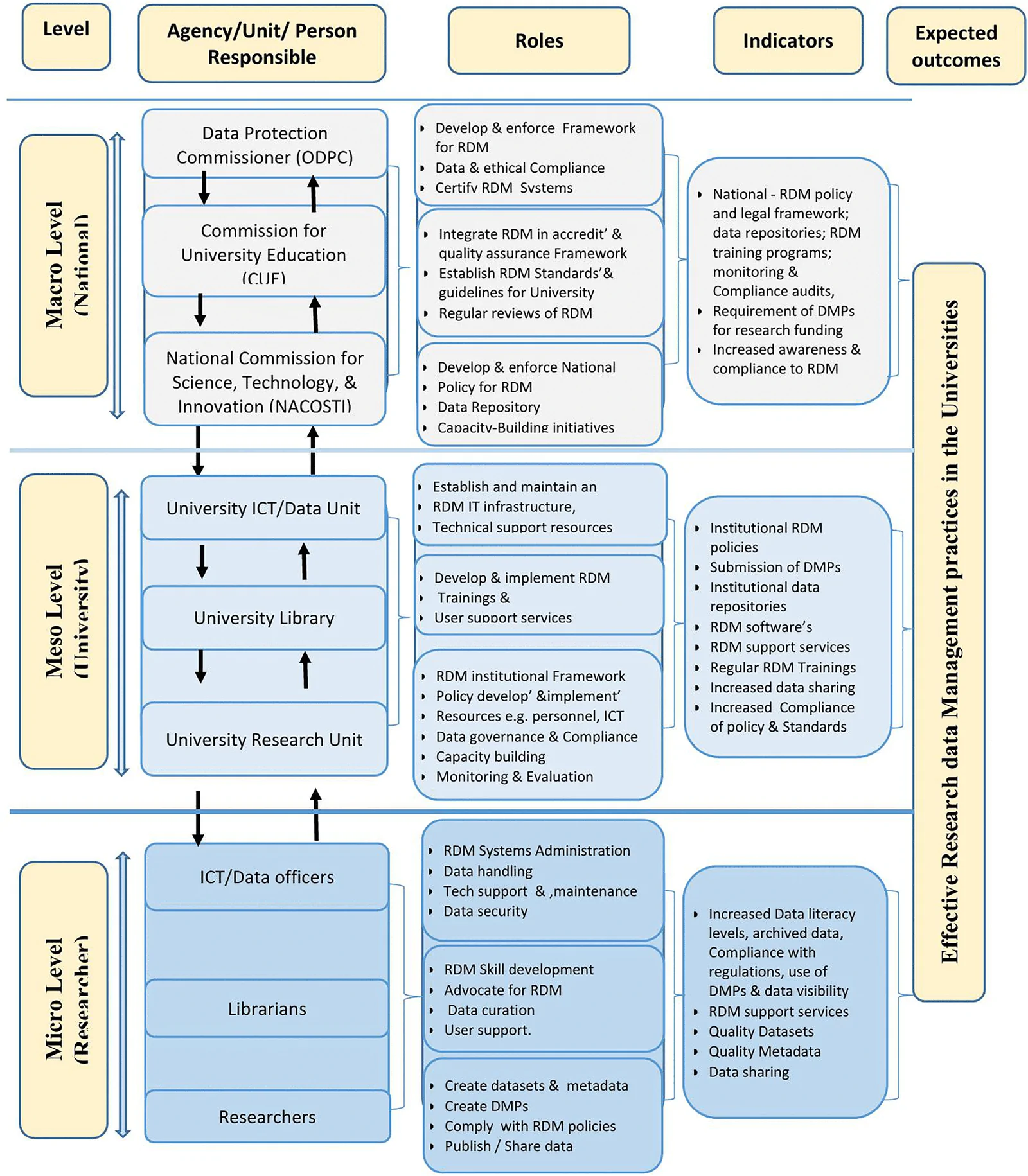
Framework for effective research data management in the universities in Kenya.

The framework is a layered approach that requires coordinated efforts across micro (individual), meso (institutional), and macro (national) levels. This design recognises that effective RDM is not solely the responsibility of individual researchers but also depends equally on institutional infrastructure and supportive national policy environments [21,48,49]. Each level plays a crucial role in the RDM ecosystem, and improvements at one level are interconnected with and enhance the effectiveness of the others. The framework uses bidirectional arrows to illustrate the dynamic relationships between individuals, institutional units, and national agencies. These visual cues highlight that effective RDM requires ongoing feedback, support, and shared responsibility across all levels. For example, researchers depend on technical and policy support from their institutions, while universities rely on national agencies to provide a clear regulatory and infrastructural pathway. This interdependence aligns with global best-practice recommendations, in which mutual reinforcement across levels ensures the systemic and sustainable implementation of RDM [39].

In the multi-level RDM framework, each actor is assigned specific and complementary responsibilities that together ensure effective and sustainable research data management.

At the individual level, researchers, librarians, and ICT/data officers play a key role in enabling RDM throughout the data lifecycle, including creation, storage, preservation, and sharing. Effective participation in these practices requires strong technical skills, appropriate tools, and a thorough understanding of data governance and ethical standards [33]. Researchers, in particular, are responsible for developing Data Management Plans (DMPs), documenting datasets with detailed metadata, and depositing data in trusted repositories, following both institutional and national policies [7,33,48]. Librarians are crucial in providing training and expertise on metadata standards, repository use, and RDM best practices. Their expanding role as data stewards positions them as vital in enhancing research data literacy and advancing open science principles [10]. Meanwhile, ICT and data officers support the technical infrastructure necessary for secure and efficient RDM. They handle the deployment, maintenance, and monitoring of data management systems, ensuring compliance with cybersecurity protocols and Kenya’s Data Protection Act (2019), while also facilitating interoperability and long-term data preservation. At the individual level, effective RDM practice indicators include increased adoption and use of Data Management Plans (DMPs), consistent deposition of research data into open repositories, and adherence to standardised metadata practices that enhance data discoverability and reuse [33].

At the institutional level, universities act as key enablers by establishing essential infrastructure, policy frameworks, and support systems for sustainable RDM. Within this structure, leadership roles are strategically shared among research offices, university libraries, and ICT departments. This approach aligns with global best practices in building institutional RDM capacity [16,39,50]. University libraries, in particular, serve as custodians of institutional repositories and play a vital role in offering researcher support services that ensure data accessibility, discoverability, and long-term preservation [22,28]. Beyond their current responsibilities, libraries can broaden their scope to include RDM-focused services such as training, metadata support, and guidance on developing Data Management Plans (DMPs). ICT departments are tasked with establishing and maintaining the technical infrastructure that underpins secure, scalable, and interoperable data management systems. Their role also includes ensuring that these systems meet cybersecurity standards and can support the entire research data lifecycle. Research offices have the role of providing strategic oversight by coordinating the development and implementation of institutional RDM policies, facilitating training programmes, and ensuring that institutional practices comply with funder requirements and ethical standards. This institutional alignment reflects the principles outlined in established models, such as the Digital Curation Centre’s RDM Lifecycle Model [32,40] and the ARDC Capability Maturity Model [39]. Both of which emphasise the importance of institutional strategy in effective RDM implementation.

At the national level, NACOSTI, CUE, and the ODPC play central roles in shaping policy, providing oversight, and strengthening the capacity for research data management (RDM). This tripartite model reflects international best practices, as seen in countries such as Australia, South Africa, and the United Kingdom, where national bodies coordinate RDM policy, infrastructure, and compliance [7,16,39,40].

NACOSTI is particularly well-positioned to spearhead the development of a national RDM policy framework, given its mandate to regulate research licensing, protect intellectual property rights, enforce data-sharing mandates, and promote open science. Drawing from South Africa’s example, where the National Research Foundation (NRF) mandates data-sharing policies, NACOSTI could incorporate data management plan (DMP) requirements into licensing procedures and advocate for the establishment of a centralised national repository to enhance data accessibility and preservation [20].

CUE, as the regulator of higher education, could integrate RDM requirements into university accreditation and quality assurance standards, ensuring that institutions develop and implement robust RDM policies as part of their broader commitments to research integrity and academic quality. Similarly, the ODPC has a vital role in ensuring compliance with Kenya’s data protection laws. Through certification and regulatory guidance, it could align institutional RDM practices with data privacy standards, akin to the enforcement mechanisms of the EU’s General Data Protection Regulation [17].

Together, these three agencies form a foundational triad capable of driving a coherent and enforceable national RDM ecosystem. Within this framework, mature national RDM systems are characterised by comprehensive policies that guide institutions, structured training programmes to build capacity, and robust auditing and compliance mechanisms to ensure adherence to standards. These elements collectively reflect the integration and maturity of RDM practices across the entire research landscape (ANDS, 2015; NRF, 2019).

The study also recommends that;

1. The universities in Kenya should institutionalise RDM by developing and implementing RDM policies that provide a structured framework for managing research data, investing in dedicated RDM IT infrastructure such as institutional repositories and data management systems, and establishing research data management support services in the library and research office, including data literacy training and technical assistance. Additionally, they should recruit or retrain librarians, ICT officers, or data officers with data management competencies to support researchers, and introduce incentives to encourage and sustain RDM efforts. These steps will shift RDM from personal, isolated efforts to coordinated institutional practices, fostering better data management, sharing, and compliance with global standards and best practices.
2. Higher Education Institutions (HEIs) in Kenya should adopt the proposed multilevel RDM framework, which delineates the roles and responsibilities of individuals, units, and relevant agencies. Further, it provides a structured approach, specifying actionable tasks for each entity, such as researchers, librarians, ICT departments, libraries, research offices, and external regulatory bodies like NACOSTI and CUE, and defines the expected outcomes for effective RDM practices.
